# Supplementary material for: Changes in Free-Living Glycemic Profiles after 12 Months of Lifestyle Intervention in Children with Overweight and with Obesity
Source: Nutrients. 2020 Apr 26;12(5):1228. doi: 10.3390/nu12051228 (PMC7282030; doi:10.3390/nu12051228)
Supplement: Supplementary file 1 [file nutrients-12-01228-s001.pdf]

Table S1. 48-hour sensor glucose measurements at baseline and after 12 months lifestyle intervention

	Baseline	% of time spent within the range	% of children reaching threshold	After 12 months intervention	% of time spent within the range	% of children reaching threshold
Time in level 2 hypoglycemic range (<3.0 mmol/L), minutes	0 (0-395)	0 (0-14)	24	0 (0-250)	0 (0-9)	27
Time in level 1 hypoglycemic range (3.0-3.9 mmol/L), minutes	65 (0-870)	2 (0-30)	73	110 (20-1270)	4 (1-44)	82
Sensor glucose ≥ 6.7 mmol/L, minutes	45 (0 - 895)	2 (0 - 31)	64	13 (0 - 210) A	1 (0 - 7)	64
Sensor glucose ≥ 7.8 mmol/L, minutes	0 (0 - 190)	0 (0 - 7)	36	0 (0 - 345)	0 (0 - 12)	27
Time in target range (3.9-10.0 mmol/L), minutes	2815 (1615-2880)	98 (56-100)	100	2770 (1545-2880)	96 (54-100)	100
Time in secondary target range (3.9-7.8 mmol/L), minutes	2705 (1615-2880)	94 (56-100)	100	2695 (1545-2880)	94 (54-100)	100
Time in level 1 hyperglycemic range (>10.0 mmol/L), minutes	0 (0-40)	0 (0-1)	6	0 (0-0)	0 (0-0)	0
Time in level 2 hyperglycemic range (>13.9 mmol/L), minutes	0 (0-0)	0 (0-0)	0	0 (0-0)	0 (0-0)	0

Data are presented as median (minimum-maximum). A = significant difference between baseline and after 12 months follow-up.

Table S2. 48-hour sensor glucose measurements at baseline and after 12 months lifestyle intervention

	Baseline	% of time spent within the range	After 12 months intervention	% of time spent within the range
Daytime: time in level 2 hypoglycemic range (<3.0 mmol/L), minutes	0 (0-190)	0 (0-11)	0 (0-85)	0 (0-5)
Daytime: time in level 1 hypoglycemic range (3.0-3.9 mmol/L), minutes	0 (0-105)	0 (0-6)	25 (0-470)	1 (0-26)
Daytime: sensor glucose ≥ 6.7 mmol/L, minutes	25 (0-665)	1 (0-37)	40 (0-685)	2 (0-38)
Daytime: sensor glucose ≥ 7.8 mmol/L, minutes	0 (0-175)	0 (0-10)	0 (0-200)	0 (0-11)
Daytime: time in target range (3.9-10.0 mmol/L), minutes	1800 (1505-1800)	100 (84-100)	1785 (1245-1800)	99 (69-100)
Daytime: time in secondary target range (3.9-7.8 mmol/L), minutes	1745 (1505-1800)	97 (84-1000)	1710 (1245-1800)	95 (69-100)
Daytime: time in level 1 hyperglycemic range (>10.0 mmol/L), minutes	0 (0-40)	0 (0-2)	0 (0-0)	0 (0-0)
Daytime: time in level 2 hyperglycemic range (>13.9 mmol/L), minutes	0 (0-0)	0 (0-0)	0 (0-0)	0 (0-0)
Nighttime: time in level 2 hypoglycemic range (<3.0 mmol/L), minutes	0 (0-350)	0 (0-32)	5 (0-260)	0.5 (0-24)
Nighttime: time in level 1 hypoglycemic range (3.0-3.9 mmol/L), minutes	50 (0-620)	5 (0-57)	50 (0-580)	5 (0-54)
Nighttime: sensor glucose ≥ 6.7 mmol/L, minutes	0 (0-290)	0 (0-27)	0 (0-490)	0 (0-45)

Nighttime: sensor glucose ≥ 7.8 mmol/L, minutes	0 (0-45)	0 (0-4)	0 (0-120)	0 (0-11)
Nighttime: time in target range (3.9-10.0 mmol/L), minutes	1030 (110-1080)	95 (10-100)	1025 (240-1080)	95 (22- 100)
Nighttime: time in secondary target range (3.9-7.8 mmol/L), minutes	1030 (110-1080)	95 (10-100)	980 (240-1080)	91 (22-100)
Nighttime: time in level 1 hyperglycemic range (>10.0 mmol/L), minutes	0 (0-0)	0 (0-0)	0 (0-0)	0 (0-0)
Nighttime: time in level 2 hyperglycemic range (>13.9 mmol/L), minutes	0 (0-0)	0 (0-0)	0 (0-0)	0 (0-0)

Data are presented as median (minimum-maximum)

Table S3. Correlation coefficients between baseline characteristics and sensor glucose measurements – subgroup analysis for the children with an increase in BMI z-score

	Δ Median sensor glucose	Δ Maximum sensor glucose	Δ Minimum sensor glucose	Δ CONGA1	Δ CONGA2	Δ CONGA4	Δ CV	Δ AUC
Δ BMI z-score	-0.302	0.195	0.045	-0.172	-0.050	-0.044	0.003	-0.177
Δ Fasting glucose	0.181	0.579	0.326	0.419	0.264	0.158	0.321	0.598 [A]
Δ Fasting insulin	0.068	0.073	-0.284	0.188	0.145	0.186	0.139	0.29
Δ HOMA-IR	0.025	0.182	0.036	0.441	0.357	0.324	0.343	0.388
Δ HbA1c	0.252	-0.320	0.243	-0.284	-0.404	-0.399	-0.439	0.354
Δ Glucose 2-h after glucose load	-0.127	0.343	0.191	0.274	0.307	0.282	0.296	0.258
Δ Total cholesterol	-0.330	-0.581 [A]	-0.278	-0.506	-0.379	-0.250	-0.473	-0.568
Δ LDL-cholesterol	-0.291	-0.580 [A]	-0.301	-0.327	-0.244	-0.111	-0.375	-0.518
Δ HDL-cholesterol	-0.427	-0.357	-0.183	-0.239	-0.134	-0.174	-0.061	-0.397
Δ Triglycerides	0.195	0.273	0.116	-0.213	-0.163	-0.171	-0.101	0.183
Δ Systolic blood pressure z-score	0.099	-0.473	0.049	-0.362	-0.471	-0.614 [A]	-0.393	0.082
Δ Diastolic blood pressure z-score	0.052	0.789 [A]	0.302	-0.552	-0.671 [A]	-0.728 [A]	-0.664 [A]	-0.104

Correlations between variables were determined by Pearson’s correlation coefficient or Spearman's correlation analysis, as appropriate. A = significant correlation.

Δ= delta; HOMA-IR = homeostatic model assessment of insulin resistance; CV = coefficient of variation; CONGA = continuous overlapping net glycaemic action; CONGA presented for 1, 2, or 4-hour time differences; AUC = area under the curve.
